# Potential Roles of the Gut Microbiota in Pancreatic Carcinogenesis and Therapeutics

**DOI:** 10.3389/fcimb.2022.872019

**Published:** 2022-04-06

**Authors:** Qiaoyu Yang, Jihang Zhang, Yin Zhu

**Affiliations:** ^1^ Department of Gastroenterology, The First Affiliated Hospital of Nanchang University, Nanchang University, Nanchang, China; ^2^ Queen Mary College, Nanchang University, Nanchang, China; ^3^ Institute of Cardiovascular Diseases, Xinqiao Hospital, Third Military Medical University, Chongqing, China

**Keywords:** gut microbiota, pancreatic cancer, carcinogenesis, diagnosis, treatment

## Abstract

The intestinal microenvironment is composed of normal gut microbiota and the environment in which it lives. The largest microecosystem in the human body is the gut microbiota, which is closely related to various diseases of the human body. Pancreatic cancer (PC) is a common malignancy of the digestive system worldwide, and it has a 5-year survival rate of only 5%. Early diagnosis of pancreatic cancer is difficult, so most patients have missed their best opportunity for surgery at the time of diagnosis. However, the etiology is not entirely clear, but there are certain associations between PC and diet, lifestyle, obesity, diabetes and chronic pancreatitis. Many studies have shown that the translocation of the gut microbiota, microbiota dysbiosis, imbalance of the oral microbiota, the interference of normal metabolism function and toxic metabolite products are closely associated with the incidence of PC and influence its prognosis. Therefore, understanding the correlation between the gut microbiota and PC could aid the diagnosis and treatment of PC. Here, we review the correlation between the gut microbiota and PC and the research progresses for the gut microbiota in the diagnosis and treatment of PC.

## Introduction

Pancreatic cancer (PC) is one of the most lethal malignancies in the world. It was the tenth most common cancer in men in 2021 and the eighth-most frequent cancer in women in the United States in 2021 (Siegel et al., 2021). PC is the seventh leading cause of cancer deaths in both sexes worldwide (Sung et al., 2021). It is difficult to detect the onset of PC, as early symptoms of PC are atypical. Many patients are diagnosed with local progression or distal metastasis and are not suitable for surgery. Thus, the 5-year survival rate is less than 10% (Siegel et al., 2021). Surgical resection is currently the only treatment option, but only 20% of PC are surgically removed at the time of diagnosis (Puckett and Garfield, 2022). Age, smoking, alcohol consumption, obesity, diabetes, genetic factors and chronic pancreatitis are considered traditional risk factors for PC (Klein, 2021). They may lead to chronic pancreatitis and further promote the occurrence of PC. However, research progress in the etiology, diagnosis and treatment of PC lags behind that of other tumors, and the etiology is still not completely clear. Currently, more evidence has suggested that the gut microbiota may be associated with the pathogenesis of PC and may be a promising therapeutic target for PC treatment.

The human gut microbiota is a group of microbes that live in the gut and is composed of more than 10^14^ microbes belonging to more than 1000 different species. The intestinal microbiota plays an important role in human physiology by influencing metabolism, regulating the mucosal immune system, promoting digestion and regulating the intestinal structure. When humans are threatened by some diseases, the gut microbiota can protect the human body by regulating inflammatory responses, providing nutrition and hormonal homeostasis, and producing bacterial metabolites with metabolic effects (Ertz-Archambault et al., 2017). The gut microbiota not only affects the digestive system but also plays an important role in many other diseases, including metabolic disease, cardiovascular disease, and central nervous system disease (Illiano et al., 2020). In recent years, some studies have shown that patients with PC may have unique oral and fecal microbiota compared to healthy individuals (Ren et al., 2017; Fan et al., 2018), suggesting that the intestinal microenvironment is involved in the occurrence and development of PC. Furthermore, pancreatic inflammation is the most important factor in inducing PC, and many immune cells cooperate to promote carcinogenesis through various mechanisms. It has been reported that macrophages are activated by some antigens of the gut microbiota and induce the interleukin-6 (IL-6)-Janus kinase 2 (Jak2)-signal transducer and activator of transcription 3 (STAT3) signaling pathway and nuclear factor-kappaB (NF-κB) signaling pathway (Lesina et al., 2011). Accumulated inflammatory responses may induce KRAS gene mutations, and the accumulation of numerous genetic mutations ultimately leads to the carcinogenesis of pancreatic cells (Ryan et al., 2014). These results indicate that PC has a certain relationship with changes in the gut microbiota. A deeper understanding of the interaction between PC and the gut microbiota is contributed to exploring the role of the gut microbiota in early diagnosis and treatment of PC.

Increasing evidence suggests that the gut microbiota is associated with PC susceptibility, initiation, and progression and may influence therapeutic efficacy. However, only radical surgery can eradicate PC, and there are few treatments currently available. Early detection and diagnosis could provide the best opportunity to enhance patient quality of life and increase the survival rate. However, to date, there are no acknowledged screening tools or biomarkers for treating PC (Wei et al., 2019). Therefore, the mechanism of the gut microbiota in PC still needs to be further explored. As research continues to deepen, gut microbiota-based diagnoses and treatments are proposed and applied, which may drive great advances in precision medicine. This paper reviews the recent research progress in the relationship between the gut microbiota and PC, the possible mechanisms involved, the impact of risk factors on the gut microbiota, and the role of gut microbiota in the early diagnosis and treatment of PC.

## Gut Microbiota

The human intestinal microecosystem contains thousands of species of bacteria and trillions of microbial cells, accounting for the vast majority of the total number of human microorganisms. It is an important and complexed microecosystem of the human body (Heintz-Buschart and Wilmes, 2018). The specific composition features of the gut microbiota vary between individuals and can be altered by many factors, including age, sex, ethnicity, diet and medications (Maffei et al., 2017; Brooks et al., 2018; Rothschild et al., 2018; Kolodziejczyk et al., 2019; Vemuri et al., 2019). Markers of microbial stability, such as richness and diversity, are often used as indicators of intestinal health because of their association with chronic diseases and metabolic dysfunction (Lee et al., 2021). The gut microbiota is involved in a variety of important physiological functions, such as digestion, absorption, energy metabolism, immune regulation and intestinal mucosal defense, by regulating neural, immune and endocrine functions (Sekirov et al., 2010). It is also affected by inheritance and the diet of the host (Belkaid and Hand, 2014; Sonnenburg and Bäckhed, 2016). The dysregulation of the gut microbiota may lead to digestive, immune, cardiovascular, respiratory, and even neurological disorders (Fan and Pedersen, 2021). The gut microbiota can degrade polysaccharides into monosaccharides and short-chain fatty acids (SCFAs) in the gastrointestinal tract (Makki et al., 2018). SCFAs are important energy sources and signaling molecules that can promote the proliferation and differentiation of intestinal epithelial cells and regulate host metabolism and the immune system. The main metabolites of SCFAs are acetate, propionate and butyrate (Koh et al., 2016). Butyrate is the main energy source of human colon cells. It acts on intestinal epithelial cells to maintain the anaerobic state of the intestine and prevents the imbalance of bacterial homeostasis by limiting the production of nitrate and oxygen (Cummings et al., 1987).

## The Relationship Between the Gut Microbiota and PC

Some epidemiological studies have suggested that the occurrence of PC is related to the dysregulation of the gut microbiota, but the exact role of the gut microbiota in the pathogenesis of PC has not been fully determined. Compared with healthy individuals, patients with PC have a variety of microbiota changes in the oral cavity, gastrointestinal tract and pancreas (Ertz-Archambault et al., 2017). Improper oral care and related diseases, such as periodontal disease and tooth loss, are risk factors for PC. Studies have shown that such diseases can lead to an imbalance of the composition and proportion of oral microbiota. In a 16-year health follow-up of 51,529 American men, Michaud et al. found 216 cases of PC (Michaud et al., 2007). After excluding the effects of risk factors such as smoking, alcohol consumption and diabetes, those with periodontal disease had a 64% increased risk of PC compared with those without periodontal disease. *Porphyromonas gingivalis* was the dominant bacteria in the oral cavity of this population (Mysak et al., 2014). The incidence of PC in periodontal disease patients with high levels of antibodies against *P. gingivalis* is twice as high as that in healthy individuals (Michaud et al., 2013). Fan et al. characterized the oral microbiota composition of 361 patients with pancreatic ductal adenocarcinoma (PDAC) and 371 healthy participants matched by age, race, gender, body mass index, alcohol consumption and smoking status, suggesting that *P. gingivalis* increased the risk of PDAC by 59% (Fan et al., 2018). Moreover, *Helicobacter pylori (H. pylori)* can also be considered a risk factor for PC and is indirectly involved in the occurrence and development of PC. Some studies have found that the incidence of *H. pylori* infection is higher in patients with PC, and the positive rate of *H. pylori* serum antibody is also higher (Wei et al., 2019). However, in chronic pancreatitis and PC, the expression of *H. pylori* DNA in pancreatic fluid or tissue could not be detected by PCR, suggesting that *H. pylori* may not directly trigger the occurrence of PC. Possible indirect mechanisms are immune escape and inflammatory reactions (Jesnowski et al., 2010). Some studies have shown that *H. pylori* can secrete cytotoxin-associated proteins and vacuolins, which promote chronic inflammatory oxidative stress and damage host DNA to trigger cellular carcinogenesis (Knorr et al., 2019).

To determine the characteristics of the microbiota in human pancreatic tumors, Pushalkar et al. conducted rRNA gene sequencing on pancreatic tumors from 12 patients by using the 16S amplification sequencing method. Analysis aligned intestinal bacterial structures of PC patients to healthy individuals. There were 13 different phyla detected in human PDAC, among which *Proteobacteria* (45%), *Bacteroides* (31%) and *Firmicutes* (22%) were the most abundant (Pushalkar et al., 2018). Additionally, the pancreas is connected to the gastrointestinal tract through the pancreatic duct, which provides conditions for translocation of gut microbiota. It is speculated that gut microbiota colonization in the pancreas is caused by duodenal or biliary bacterial reflux (Pushalkar et al., 2018). Thomas et al. reported that both normal and pancreatic tumor tissues have their own microbiome, but their microbiome could not be distinguished between disease states. Additionally, the presence of gut microbes combined with oral and gut microbiota translocations accelerates the progression of PC (Thomas et al., 2018).

## Mechanisms by Which the Gut Microbiota Causes PC

### Inflammatory Responses

**Figure 1:**
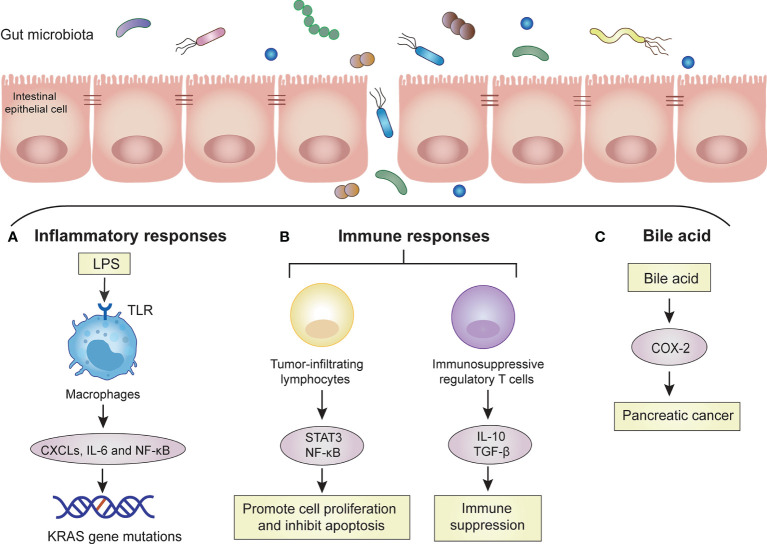
Mechanisms of the gut microbiota in PC. The gut microbiota promotes the carcinogenesis of PC cells by these mechanisms, such as inflammatory response, immune response and bile acid. Dysbiosis of gut microbiota increases bacterial invasion, which promotes carcinogenesis of pancreatic cells. **(A)** Inflammatory responses: Invasive bacteria enter the bloodstream and release large amounts of LPS. LPS is recognized by TLRs on macrophages. Macrophages are activated to release some proinflammatory cytokines (CXCLs, IL-6) and activate signaling pathways (NF-kB). Accumulated inflammatory responses induce KRAS gene mutation. **(B)** Immune responses: Invasive bacteria activate tumor-infiltrating lymphocytes and immunosuppressive regulatory T cells. Tumor-infiltrating lymphocytes can promote cell proliferation and inhibit apoptosis through the STAT3 and NF-κB signaling pathways. Immunosuppressive regulatory T cells lead to systemic immune suppression by releasing IL-10 and transforming growth factor-β (TGF-β). **(C)** Bile acid: bile acid can upregulate the mRNA of cyclooxygenase-2 (COX-2) in PC cells to increase the invasion ability of tumor cells.

The inflammatory response is the defense reaction produced by the body to resist external infection and tissue damage, but an excessive inflammatory response can lead to tissue cell cancer. Some studies have shown that chronic inflammation caused by the gut microbiota or their metabolites may lead to tumor production (Kundu and Surh, 2008; Pesic and Greten, 2016). Pancreatic carcinogenesis may be promoted by both remote and local pathways. Some high-risk factors for PC (unhealthy lifestyle, obesity, diabetes and chronic pancreatitis) can interact with the gut microbiota. These interactions can cause metabolic pathways to activate proinflammatory pathways and destroy the intestinal barrier. The metabolites of the gut microbiota may enter the pancreas through the mesenteric vein route, while harmful bacteria enter the pancreas through the mesenteric lymphatic route. These transitions can promote the differentiation of inhibitory immune cells, thereby affecting the tumor microenvironment (Thomas and Jobin, 2020). Some high-risk factors (smoking and alcohol consumption) can interact with the oral microbiota, resulting in oral microbiota disorders that can cause systemic inflammation (Chang et al., 2016). After the tissue barrier is damaged, harmful bacteria in the oral cavity translocate and colonize the pancreas (Michaud et al., 2013). Through direct local action, they may cause tissue inflammation, increase the recruitment of proinflammatory cells and cytokine secretion, lead to oxidative stress imbalance in the microenvironment, and damage DNA. Thus, it ultimately results in molecular alteration and tumor transformation. Moreover, when the inflammatory response leads to the abnormal secretion of pancreatic fluid and disrupted gut microbiota, intestinal content reflux through the main pancreatic duct may cause gut microbiota to enter the pancreas. This could destroy the normal microenvironment of the pancreas (Zambirinis et al., 2014). The KRAS mutation is present in 90% of PC cases, but KRAS activation still requires the overstimulation of lipopolysaccharide (LPS)-driven inflammation (Daniluk et al., 2012). Gram-negative bacteria in the gut produce LPS that specifically binds to host Toll-like receptor (TLR), causing systemic inflammation and oncogenesis (Ochi et al., 2012). When the two are combined, inflammatory responses are activated, and the recruitment of proinflammatory cells and the secretion of cytokines are increase. The inflammatory response can promote the expression of CXC receptor 2 (CXCR2), CXC ligands (CXCLs), STAT3, and IL-6 and activate NF-κB (Zambirinis et al., 2014). In addition to the role of proinflammatory cytokines, several molecular alterations, such as oncogene mutations, tumor suppressor gene inactivation, and chromosomal and microsatellite instability, are also involved in inflammation-mediated carcinogenesis (Humphris et al., 2014; Wei et al., 2019). These studies confirm the view of many researchers that the dysbiosis of the gut microbiota leads to chronic inflammation and then subsequently to tumors (**Figure 1**).

### Immune Responses

Immune cells along with large numbers of microbiota maintain symbiotic relationships between human bodies and intestinal microbes (Mowat and Agace, 2014). There are complex interactions between the human immune system and gut microbiota. The immune system can influence the intestinal microbiota by forming a mucus barrier and secreting antibacterial proteins and immunoglobulin A (IgA). The gut microbiota can also affect local intestinal immunity, T cell development and immune system maturation by producing a variety of antigens and metabolites (Hooper et al., 2012). The intestinal microbes and their metabolites are critical to maintaining immune balance, and changes in the gut microbiota disrupt this balance. This disruption mainly affects intestinal mucosal immunity and the systemic immune response (Forbes et al., 2016). Intestinal dysbiosis does not directly interfere with the cell cycle to activate cancer signaling pathways, but it activates the immune system through multiple pathways, including tumor-infiltrating lymphocytes and their associated cytokines, innate immune cells and TLRs (Pagliari et al., 2018; Pushalkar et al., 2018). Moreover, tumor-infiltrating lymphocytes can produce proinflammatory mediators that promote cell proliferation and inhibit apoptosis through the STAT3 and NF-κB signaling pathways (Francescone et al., 2014). However, PC is often in the relatively advanced stage when the tumor microenvironment is flooded with many immune regulatory cells, and thus, the antitumor immune response is inhibited (Feig et al., 2012). For instance, immunosuppressive regulatory T cells and myeloid cells are recruited into the tumor stroma by the cytokine granulocyte-macrophage colony-stimulating factor produced by tumor cells, resulting in a disruption of T cell-mediated antitumor immunity (Pylayeva-Gupta et al., 2012). Similarly, high levels of immunoregulatory cytokines, such as IL-10 and transforming growth factor-β (TGF-β), were detected in the peripheral blood of patients with PC, suggesting systemic immune suppression in patients with PC (Bellone et al., 1999). Therefore, the immunosuppressive local microenvironment of PC not only promotes tumor progression but also leads to immunotherapy failure (**Figure 1**).

### Bile Acids

The gut microbiota participates in the catabolic process of human bile acids. Bile acids are a kind of regulatory factors that are involved in signal transduction and affect the occurrence and development of cancer through oxidative stress and chronic inflammation. This intestinal microenvironment-bile acid-PC network shows a close relationship among these three (Feng and Chen, 2016). In recent years, studies have found that bile acids can be used as signaling molecules to participate in cell signal transduction and affect tumor formation and apoptosis through oxidative stress and chronic inflammatory reactions (Houten et al., 2006). Tucker et al. showed that bile acid can upregulate the mRNA of cyclooxygenase-2 (COX-2) in PC cells to increase the invasion ability of tumor cells and speculated that bile acid is involved in the invasion process of PC (Tucker et al., 2004). The structure of bile acids allows them to act as decontamination agents that destroy the lipid molecular bilayer of the contacted cells and help carcinogens enter the cells. Bile acid can change the microvilli structure on the surface of PC cells and affect the function of mitochondria in cancer cells. Therefore, it is speculated that bile acid can affect the proliferation and invasion of PC cells through cytotoxic effects (Lu et al., 2000; Shrader et al., 2021). The gut microbiota is involved in the catabolism of bile acids, which inhibits the conversion rate of primary bile acids to secondary bile acids. This leads to a reduction in secondary bile acid content and thus reduces intestinal toxicity and the occurrence of tumors (Zampa et al., 2004) (**Figure 1**).

## Risk Factors for PC in Relation to the Gut Microbiota

### Diet and Lifestyle Habits

In addition to correlations with age, sex, genetics and other factors, existing studies have confirmed that poor diet and lifestyle habits, such as alcohol consumption and smoking, are also closely related to the occurrence of PC (Yadav and Lowenfels, 2013). These risk factors significantly affect the metabolism and microbiota composition of microbes. They can increase the proportion of harmful bacteria and decrease the proportion of beneficial bacteria in the intestine. Studies have shown that drinking large amounts of ethanol disrupts the normal gut microbiota. *Verrucomicrobia*, *Actinobacteria* and *Proteobacteria* were enriched in the intestinal tract of alcohol-addicted mice (Chen et al., 2015; Borrelli et al., 2018; Grander et al., 2018). Clinical studies also revealed that long-term alcohol consumption reduced the abundance of *Firmicutes* and *Lactobacillus* in the gut, while *Enterococcus* was significantly increased. In addition, long-term alcohol intake can also lead to bacterial overgrowth and dysbiosis in the small and large intestine (Eslamparast et al., 2014; Goodrich et al., 2014). Acetaldehyde, one of the important metabolites of ethanol, has been shown to form superoxide, acetaldehyde complexes and other toxic metabolites. These toxic metabolites not only lead to pancreatic secretion imbalance but also induce interstitial fibrosis and promote the release of various inflammatory mediators, thus leading to chronic pancreatitis (Naderian et al., 2017). The pancreatic duct is connected to the duodenum through the duodenal papilla. Poor diet and lifestyle habits will lead to the abnormal secretion and composition of pancreatic juice. This may lead to harmful microbiota in the intestine retrograding into the pancreatic duct, leading to abnormal changes in the microenvironment in the pancreatic duct and pancreas. An abnormal microenvironment existing in the pancreas for a long time may lead to inactivation of tumor suppressor genes and abnormal activation of oncogenes and ultimately induce PC (Hussain et al., 2003).

### Obesity

Obesity is an important risk factor for the development of PC. Epidemiological surveys have indicated that the incidence of PC is significantly higher in wealthy areas where the fat content is higher in the daily diet than in gradually developing areas (Vincent et al., 2011). In a study of obesity and the gut microbiota, germ-free mice gained weight when bacteria from obese patients were transplanted (Fei and Zhao, 2013). In addition to mouse models, long-term body weight gain (over 10 years) in humans was also demonstrated to be associated with low microbiota diversity, and this association was exacerbated by low dietary fiber intake. The amount of *Bacteroidetes* in the gut microbiota of obese patients was significantly reduced, while the total amount of Gram-positive *Firmicutes* in the intestinal tract of obese patients was 20% higher than that of normal adults (Armougom et al., 2009; Turnbaugh, 2012). Dysregulation of the gut microbiota may contribute to diet-induced obesity and metabolic complications through a variety of mechanisms, including immune dysregulation, altered energy regulation, altered intestinal hormone regulation, and proinflammatory mechanisms (Eibl and Rozengurt, 2019; Lee et al., 2020). In addition, adipose tissue can release a variety of inflammatory factors, such as C-reactive protein (CRP), tumor necrosis factor-α (TNF-α), IL-6, and especially monocyte chemoattractant protein-1 (MCP-1), to promote the infiltration of inflammatory cells in fat. These factors promote inflammation in the body, which may induce normal cell carcinoma in the body (Carvalho et al., 2012; Piya et al., 2013). Moreover, obesity can also reverse the intestinal microecology and influence the gut microbiota composition (Hakkak et al., 2017), which indirectly reflects the correlation between the gut microbiota and PC.

### Diabetes

Many studies in recent years have suggested that diabetes is closely associated with PC. Type 2 diabetes is often accompanied by moderate intestinal dysbiosis, which is characterized by increased numbers of *Lactobacillus*, *Bacteroidetes* and *Proteobacteria* and reduced abundance of *Faecalibacterium prausnitzii* and *Firmicutes (* Craciun et al., 2022 *)*. The gut microbiota affects diabetes by affecting body mass, bile acid metabolism, chronic inflammation, insulin resistance and gut hormone regulation (Ren et al., 2017). A meta-analysis has found that the relative risk of PC in patients with a history of diabetes of less ≥2 years, ≥5 years and ≥10 years is 1.64, 1.58 and 1.50, respectively, revealing the close correlation between diabetes and PC (Song et al., 2015). Larsen et al. found that the proportion of *Betaproteobacteria* in the intestinal tract of patients with diabetes was increased, which indicated an increase in the number of Gram-negative bacteria and LPS (Larsen et al., 2010). When the intestinal mucosal barrier dysfunction is due to gut microbiota imbalance, LPS is more likely to be absorbed into peripheral blood and activate a series of inflammatory factors, such as CXCLs, CXCR2 and IL-6. These factors can induce carcinogenesis under the stimulation of a continuous inflammatory response (Cruz et al., 2013). In addition, cancer cells utilize energy mainly through glycolysis, so the high blood sugar levels of patients with diabetes may increase the proliferation of cancer cells. Hyperglycemia also affects the secretion and release of insulin and activates insulin-like growth factor 1 (IGF-1), which may promote the occurrence and development of tumors (Zhang et al., 2018). Moreover, the decreased immune function in patients with diabetes affects the antitumor effects of probiotics, such as *Bifidobacterium* (Komura et al., 2010; Sivan et al., 2015). Therefore, these effects may also be one of the factors that induce the development of PC in diabetes.

### Chronic Pancreatitis

Chronic pancreatitis is a major risk factor for PC. It is the progressive inflammation of the pancreas, which can lead to the loss of pancreatic fibrotic acinar and islet cells. Approximately 5% of patients with chronic pancreatitis can develop PC, and the risk of PC in patients with chronic pancreatitis is 13 times higher than that in the general population (Raimondi et al., 2010). A small number of studies have shown that the diversity of the gut microbiota in patients with chronic pancreatitis is significantly reduced (Zhou et al., 2020; Wu et al., 2021). The relative abundance of *Faecalibacterium prausnitzii* and *Ruminococcus bromii* was decreased in patients with chronic pancreatitis, especially in patients with diabetes (Jandhyala et al., 2017). Moreover, approximately 36% of patients with pancreatitis develop excessive growth of the gut microbiota, which is called small intestine bacterial overgrowth (SIBO). SIBO is a malabsorption syndrome that causes excessive fermentation and inflammation in the small intestine (Gasbarrini et al., 2011; Capurso et al., 2016). Some patients with chronic pancreatitis who carry KRAS gene mutations have an increased cancer risk. SIBO may lead to KRAS gene mutations through the LPS-driven inflammatory response and TLR-mediated NF-κB signaling pathway, thus accelerating the development of PC (Huang et al., 2014). LPS and TLR interact and induce chronic inflammation. TLR expression on various immune cells enables immune cells to recognize a variety of microbial-related molecules and noninfectious inflammatory injury-related molecules and activates the NF-κB and mitogen-activated protein kinases (MAPK) signaling pathways to persistently induce pancreatic inflammation (Meng et al., 2018). Moreover, pancreatitis and KRAS gene mutations are two common factors in PC, both of which are essential for pancreatic intraepithelial neoplasia (PanIN) and invasive cancer in animal models (Guerra et al., 2007).

## The Gut Microbiota and the Diagnosis and Prognosis of PC

Currently, the traditional diagnostic methods for PC are mainly dependent on signs, symptoms, tumor markers, imaging manifestations, and histopathological or cytological tests (Chu et al., 2017). However, positive results cannot be detected until the onset of PC, even in the middle and late stages. The progression of PC is through PanIN of the pancreas during carcinogenesis. Although these changes in pancreatic morphology are histologically different, imaging techniques used in clinical practices are unable to distinguish early PanIN from the normal pancreas (Hruban et al., 2000). With a deeper understanding of intestinal microecology, some studies have demonstrated that intestinal microecological changes may provide new biological markers for the diagnosis of PC. The experiment by Torres et al. showed that compared with normal people, the proportion of *Leptotrichia* increased and the proportion of *Porphyromonas* decreased in the saliva of PC patients, suggesting that the proportion of the two may be used as a biological marker of PC (Torres et al., 2015). Some intrapancreatic bacteria, such as *H. pylori*, were differentially increased in the pancreas of patients with PC. If human pancreatic cells are infected with *H. pylori*, they can colonize the pancreas. The colonization of *H. pylori* is associated with the activation of molecular pathways related to the occurrence and maturation of PC (Takayama et al., 2007), thus contributing to the malignancy of PC (Nilsson et al., 2006). In addition, oral *Fusobacterium* has been shown to reduce the risk of PC, and *Fusobacterium* colonization was considered to be an independent prognostic factor that significantly decreased the survival rate of PC (Mitsuhashi et al., 2015). With the rapid development of medicine, the early analysis of the gut microbiota may provide an important basis for the early diagnosis and treatment of PC.

Riquelme et al. found that the gut microbiota can determine the survival of PC patients (Riquelme et al., 2019). They elucidated the role of the tumor microbiota and immune system in the long-term survival (LTS) of PC patients. They found high alpha-diversity in the tumor microbiota of patients with LTS compared with patients with short-term survival (STS). They also identified four microbial signatures in the tumor microbiota (*Pseudoxanthomona*, *Saccharopolsypora*, *Streptomyces* and *Bacillus clausii*) that can predict long-term survival in patients with PC. Using fecal microbiota transplantation from STS, LTS, or control donors, they found that the tumor microbiota could be differentially regulated and affect tumor growth and tumor immune infiltration (Riquelme et al., 2019). Fecal microbiota transplantation can significantly improve the prognosis of patients with PC, and manipulating the microbiota could be used as a treatment means. Although PDAC has the same mortality, there is heterogeneity in the clinical symptoms and limited genomic variation. A highly aggressive tumor subtype called “basal-like” was identified in PC. This subtype shows a poor prognosis and a higher inflammatory response. Metagenomic analysis of pancreatic tumors from 62 different clinical subtypes showed that “basal-like” PDAC was characterized by active antimicrobial immune and inflammatory responses and had different microbiota characteristics. These bacteria have the potential to induce inflammation and are associated with the expression of cancer genes. This study confirmed that the tumor microbiota is closely related to the carcinogenesis and inflammatory induction of PC. Thus, these findings demonstrate the predictive value of the microbiota, and introduce new ideas for the treatment and intervention of diseases (Guo et al., 2021).

## The Role of the Gut Microbiota in Prevention and Treatment of PC

Although surgery, chemotherapy, and biological therapy for the treatment of PC have made some progress, these treatment measures are costly. They also cause great damage to the patient’s body, and the survival rate is still low. Therefore, there is an urgent need to find a new clinical treatment for PC. The study of the gut microbiota can provide a new direction for the prevention and treatment of PC. Consequently, fecal microbiota transplantation, probiotics, butyric acid, immunotherapy, and other methods may represent new therapies to treat or prevent the development of PC.

### Fecal Microbiota Transplantation

**Figure 2:**
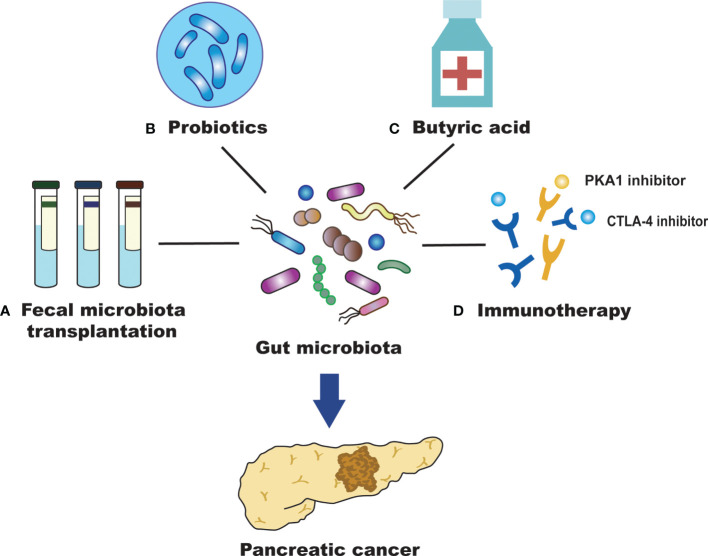
Efficient treatment for PC by targeting the gut microbiota. **(A)** Fecal microbiota transplantation (FMT) may inhibit the development of pancreatic cancer (PC) by regulating the intestinal microbiota, reducing the production of inflammatory mediators and cytotoxic metabolites, and improving the dysregulation of intestinal microbiota. **(B)** Probiotics may alter gut microbiota and affect the survival of patients with PC. They can also promote human health with antitumor effects and maintain the homeostatic state of intestinal microorganisms. **(C)** Butyric acid can promote the differentiation of PC cells and inhibit their invasion and metastasis of PC cells. **(D)** The gut microbiota can promote anti-PC immunotherapy by regulating immune checkpoints (PD-L1 and CTLA-4).

Fecal microbiota transplantation (FMT) refers to the process of transferring the gut microbiota and its metabolites from a healthy donor to the recipient. This is currently the best way to rebuild the intestinal microecology and treat intestinal and extraintestinal diseases (**Figure 2**) (Wang et al., 2019). FMT has attracted increasing attention due to its therapeutic efficacy and convenience. A variety of diseases can benefit from the treatment of FMT, including *Clostridium difficile* infection, inflammatory bowel disease, obesity, and cancer (Smits et al., 2013; Chen et al., 2019). FMT may inhibit the development of PC by regulating the gut microbiota, reducing the production of inflammatory mediators and cytotoxic metabolites, and improving the dysregulation of gut microbiota (Bibbò et al., 2017). High abundance of *Fusobacterium* in PC tissue correlates with poor prognosis (Mitsuhashi et al., 2015), suggesting that *Fusobacterium* could become a promising prognostic parameter after the FMT treatment of PC. In another FMT study applying to PC, stool samples from patients with advanced PC, patients with PC who had survived more than five years, or healthy controls were transplanted into mice. After 35 days, the tumors of mice treated with FMT from the advanced PC group were found to be much larger than those treated with FMT from patients with PC who had survived more than five years or healthy controls (Riquelme et al., 2019). It has been suggested that the diversity of the gut microbiota in patients with PC can affect the development of tumors; thus, FMT may become a new treatment for PC in the future. However, there are still few studies on the application of FMT in the treatment of PC. More animal studies are needed to demonstrate the efficacy and safety of FMT and to explore its role in clinical treatment. In addition, we also need to develop therapeutic strategies for gut microbiota with different functions, improve transplant matching, and enhance the effect of personalized FMT treatment for PC.

### Probiotics

Probiotics have the role of promoting human health, maintaining the homeostasis of intestinal microbes, alleviating the inflammatory response, regulating the immune system, and promoting antitumor efficiency to inhibit PC progression (Meng et al., 2018). Probiotics can not only form a membrane barrier by adhering to intestinal epithelial cells and competitively inhibiting the growth of pathogenic bacteria but also secrete antibacterial substances to inhibit the colonization and proliferation of pathogenic bacteria. *Bifidobacterium* is an important intestinal probiotic that can inhibit tumor development and promote the high expression of some tumor-suppressive metabolites. These effects may possibly act *via* mechanisms that prevent harmful bacteria from colonizing the intestinal tract. This would produce acidic substances and reduce the pH of the microenvironment to maintain the intestinal microecological balance. The metabolism of gut bacteria can affect cancer treatment, including chemotherapy and immunotherapy. Although not directly used in PC, oral *Bifidobacterium* operational taxonomic unit OTU_681370 combined with anti-programmed cell death-ligand 1 (PD-L1) immunotherapy can almost completely inhibit the growth of mouse melanoma. It can also promote the infiltration of T cells into the tumor microenvironment, regulate the activation of cytokine receptors to produce interferon-γ (INF-γ) and then promote the growth of monocytes (Sivan et al., 2015). Therefore, establishing a healthy gut microbiota and inhibiting the growth of pathogenic bacteria, in combination with chemical therapy and immune therapy, may improve the survival time of patients with PC. The most commonly used probiotics in the clinic are *Lactobacillus* and *Bifidobacterium*, which can inhibit the growth of *Enterobacteria*. *Bifidobacterium* can stimulate the immune system to attack tumor cells and enhance the efficacy of cancer immunotherapy (Sivan et al., 2015). Moreover, probiotics can regulate the composition of the microbiota and reduce the side effects, such as diarrhea and mucositis, caused by chemotherapeutic drugs (Serna-Thomé et al., 2018). However, further clinical trials are needed to confirm the role of probiotics in the prevention and treatment of PC (**Figure 2**).

### Butyric Acid

Studies have shown that butyrate is closely related to the occurrence and development of tumors (Bolden et al., 2006). At present, there are numerous studies on the antitumor effect of butyric acid in lung cancer, bowel cancer, bladder cancer, leukemia and so on, but there are relatively few reports on this in PC. Butyric acid plays a key role in maintaining intestinal health. It is not only the main energy source of colon epithelial cells but also an important anti-inflammatory metabolite of the gut microbiota (Canani et al., 2011; Vital et al., 2014). Sodium butyrate inhibits the invasion and metastasis of PC cells by downregulating the expression of integrin β4 (Farrow et al., 2003). It also promotes the differentiation of PC cells and induces the expression of some tumor-related antigens (Corra et al., 1993). These results suggest that butyric acid has a significant inhibitory effect on the occurrence, development and invasion of PC. *Bifidobacterium* can ferment glucose to produce acetic acid, butyric acid and lactic acid, which are SCFAs that are beneficial to intestinal health. Butyrate can achieve an antitumor effect by inhibiting histone deacetylase and telomerase activity (Tan et al., 2014). In addition, butyrate induces Treg cell amplification, produces anti-inflammatory cytokines, reduces DNA oxidative damage, induces apoptosis that already underwent DNA damage, and blocks tumor cell growth (Smith et al., 2013). These results imply that regulating butyric acid levels through the intervention of the gut microbiota may be beneficial to the prevention and treatment of PC (**Figure 2**).

### Immunotherapy

Immunotherapy is a new therapeutic concept that can control tumor growth and metastasis and even eliminate tumors by activating the body’s continuous immune response to the tumor while maintaining the functional integrity of normal tissues (Kraehenbuehl et al., 2022). In recent years, the important role of the microbiota in immune regulation and tumorigenesis has become increasingly prominent, and regulation of the microbiota is important for the immunotherapy of PC (Sethi et al., 2019). Some studies confirmed that microbes can strengthen the therapeutic effect of cancer immunotherapy (Leslie, 2015). The gut microbiota regulates immunosuppressive molecules that inhibit lymphocyte function and help cancer cells escape. These effects have a positive impact on the immunotherapy of PC. The gut microbiota also promotes anti-PC immunotherapy by regulating immune checkpoints (Elkrief et al., 2019). As immunosuppressive molecules, immune checkpoints can inhibit the function of lymphocytes and assist the immune escape of tumor cells (Horai et al., 2015). It has been found that as an immune checkpoint inhibitor, the cytotoxic T-lymphocyte-associated protein 4 (CTLA-4) monoclonal antibody is dependent on the gut microbiota in the treatment of tumors and cannot produce effective antitumor efficacy in the absence of the gut microbiota (Vétizou et al., 2015). In animal experiments, the gut microbiota enhanced animal responses to CTLA-4 inhibitory antibodies and stimulated the immune system to attack tumors. Researchers describe *Bifidobacterium* as immune assistants. The efficacy of PD-L1-blocking tumor antibodies were improved in mice fed probiotics containing a variety of *Bifidobacterium* (Leslie, 2015). The gut microbiota can regulate the T cell-mediated antitumor immune response by regulating the function of dendritic cells. Therefore, the selection of specific immunotherapies according to the different intestinal microecological conditions is helpful for the prevention and treatment of PC. Finding new therapeutic targets for PC and developing effective immunotherapy strategies will be a substantial long-term task that will require continuous research and exploration (**Figure 2**).

## Conclusion

The gut microbiota is a large and complex system that plays an important role in the regulation of the occurrence and development of PC. The gut microbiota studies have revealed the underlying mechanisms, predicted the risk of PC, and provided a new approach for the diagnosis and prognosis of PC. Important quoted human studies including experimental methods, microbial change and author’s conclusions are summarized in **Table 1**. The study of the mechanisms that connect the gut microbiota and PC provides a theoretical basis for subsequent prevention and therapeutic interventions. Probiotics can be combined with chemotherapy and immunotherapy to provide promising therapeutic methods for patients with PC. With the continuous development of metagenomics and immunogenomics, scientists have made breakthroughs in the application of the microbiome in immunotherapy for gastrointestinal cancer. To better apply the microbiome in the future, we need to monitor changes in each stage of the progression of PC and investigate more approaches targeting the cancer-related microbiome to improve therapeutic efficacy. The involvement of intestinal microecology in various physiological functions of the body is of great scientific value. Therefore, the mechanism of its action needs to be further explored in future research. In conclusion, it is imperative to resolve the difficulty of the early diagnosis and treatment of PC. It is necessary not only to explore the molecular biological mechanisms regulating the gut microbiota but also to continuously explore new therapeutic targets to improve the survival time and quality of life of patients with PC. Better yet, personalized treatments can be tailored to each patient based on their respective microbiome.

**Table 1:** Overview of the major gut microbiota studies involving pancreatic cancer.

Study	Study design	Microbiome specimen	Detection method	Microbial change	Author conclusion
Michaud et al. (Michaud et al., 2013)	Prospective cohort study	Plasma	Antibiotics to oral bacteria	↑*Porphyromonas gingivalis* (ATTC 53978)	Twofold higher risk of PC among individuals with high levels of antibodies against *P. gingivalis*
Fan et al. (Fan et al., 2018)	Case-control	Oral	16S sequencing	↑*Porphyromonas gingivalis* and *Aggregatibacter actinomycetemcomitans*, ↓*Fusobacteria* and *Leptotrichia*	Presence of oral microbiota are related to increased risk of PC. Decreased relative abundance of *Fusobacteria* and *Leptotrichia* are associated with subsequent risk of PC
Jesnowski et al. (Jesnowski et al., 2010)	Case-control	Pancreas	16S sequencing	NA	*H. pylori* cannot trigger PC directly
Armougom et al. (Armougom et al., 2009)	Case-control	Feces	qPCR	↑*Lactobacillus*,↓*Bacteroidetes*, ↑*Methanobrevibacter smithii*	*Lactobacillus* increases in some obese patients and *M. smithii* increases in anorexic patients
Larsen et al. (Larsen et al., 2010)	Case-control	Feces	qPCR	↑*Betaproteobacteria*, ↓*Firmicutes* and *Clostridia*	Patients with type 2 diabetes is associated with compositional changes in gut microbiota
Jandhyala et al. (Jandhyala et al., 2017)	Case-control	Feces	16S sequencing	↓*Faecalibacterium prausnitzii* and *Ruminococcus bromii*	The gut microbiota altered in patients with chronic pancreatitis, especially in patients with diabetes
Torres et al. (Torres et al., 2015)	Cross-sectional	Salivary	16S sequencing	↑*Leptotrichia*, ↓*Porphyromonas*	The ratio of *Leptotrichia* to *Porphyromonas* in the saliva may be used as a biological marker of PC
Nilsson et al. (Nilsson et al., 2006)	Case-control	Pancreas	DNA sequencing	NA	Gastric and enterohepatic *Helicobacter* species suggest a possible role in the development of chronic pancreatitis and PC
Mitsuhashi et al. (Mitsuhashi et al., 2015)	Case-control	Pancreas	qPCR RT-PCR	NA	*Fusobacterium* species may be a prognostic biomarker of PC
Riquelme et al. (Riquelme et al., 2019)	Cohort study	Pancreas	16S sequencing	↑Alpha diversity; ↑Saccharopolyspora, Pseudoxanthomona, Streptomyces, Bacillus clausii	The tumor microbiome diversity can determine the survival of PDAC patients
Sivan et al. (Sivan et al., 2015)	Pilot	Feces	16S sequencing	↑*Bifidobacterium* (OTU_681370)	*Bifidobacterium* promotes T-cell mediated antitumor immunity and activates anti-PD-L1 efficacy

PC, pancreatic cancer; PDAC, pancreatic ductal adenocarcinoma; NA, not applicable; qPCR, quantitative PCR; RT-PCR, reverse transcription PCR; OTU, operational taxonomic unit; PD-L1, programmed cell death-ligand 1. ↑: number increased; ↓: number decreased.

## Author Contributions

QY wrote the manuscript; JZ revised the manuscript; YZ provided advice. All authors contributed to the article and approved the submitted version.

## Funding

This study was supported by the National Natural Science Foundation of China (No. 81960128 and No. 8187359), and the Key Research and Development Program from the Science and Technology Department of Jiangxi Province (No. 20192ACBL20037).

## Conflict of Interest

The authors declare that the research was conducted in the absence of any commercial or financial relationships that could be construed as a potential conflict of interest.

## Publisher’s Note

All claims expressed in this article are solely those of the authors and do not necessarily represent those of their affiliated organizations, or those of the publisher, the editors and the reviewers. Any product that may be evaluated in this article, or claim that may be made by its manufacturer, is not guaranteed or endorsed by the publisher.

## Abbreviations

PC, Pancreatic cancer; IL-6, interleukin-6; STAT3, signal transducer and activator of transcription 3; NF-κB, nuclear factor-kappaB; SCFAs, short chain fatty acids; PDAC, pancreatic ductal adenocarcinoma; H. pylori, Helicobacter pylori; LPS, lipopolysaccharide; TLR, Toll-like receptor; CXCR2, CXC receptor 2; CXCLs, CXC ligands; IgA, immunoglobulin A; TGF-β, transforming growth factor-β; COX-2, cyclooxygenase-2; IGF-1, insulin-like growth factor 1; SIBO, small intestine bacterial overgrowth; PanIN, pancreatic intraepithelial neoplasia; LTS, long-term survival; STS, short-term survival; FMT, Fecal microbiota transplantation; PD-L1, programmed cell death-ligand 1; INF-γ, interferon-γ; CTLA-4, cytotoxic T-lymphocyte-associated protein 4; MAPK, mitogen-activated protein kinases; CRP, C-reactive protein; TNF-α, tumor necrosis factor-α; MCP-1, monocyte chemoattractant protein-1.
